# Introduction of the One-Minute Preceptor Model for Teaching Pathology Postgraduate Students

**DOI:** 10.7759/cureus.113833

**Published:** 2026-08-02

**Authors:** Vandana Bhatti, Roma Isaacs, Monika Sharma

**Affiliations:** 1 Department of Pathology, Department of Medical Education, Christian Medical College and Hospital, Ludhiana, IND; 2 Department of Blood Transfusion, Employees’ State Insurance Corporation Medical College and Hospital, Ludhiana, IND; 3 Department of Pediatrics, Department of Medical Education, Christian Medical College and Hospital, Ludhiana, IND

**Keywords:** omp, pathology, postgraduate, s: one minute preceptor, teaching method

## Abstract

Background: A one-minute preceptor (OMP) is a time-effective teaching method commonly used in clinical settings and is a preferred teaching method by students. It comprises five micro skills with a recent addition of a sixth micro skill. It has been utilized less commonly in paraclinical departments, with occasional studies from Anatomy and Pathology.

Methodology: This prospective cross-sectional study was conducted in the Department of Pathology, Christian Medical College and Hospital, Ludhiana, for a period of three months. It included 11 postgraduate pathology residents and seven faculty members who were sensitized to OMP followed by the conduction of OMP sessions. Perceptions of postgraduate students and faculty regarding OMP were recorded using Google Forms on a five-point Likert scale. The satisfaction index was calculated for the perceptions of postgraduate students and faculty. In-depth interviews were also conducted with postgraduate residents and faculty.

Results: All residents (11/11, 100%) and faculty members (7/7, 100%) reported that they first learned about the One-Minute Preceptor (OMP) during the introductory lecture of this study, and all residents (11, 100%) and faculty members (7, 100%) enjoyed slide-based teaching using this method. All residents (11, 100%) agreed that OMP helped build confidence, improved presentation, analytical, and reasoning skills in slide diagnosis, provided constructive feedback that enhanced learning, motivated self-directed learning, and was less threatening than the traditional slide-teaching method. Ten residents (10, 90.9%) agreed that OMP should be incorporated into regular teaching. All faculty members (7, 100%) agreed that OMP increased interaction between faculty and residents, improved residents' analytical skills, provided opportunities for feedback, and should be adopted as a regular teaching method.

Conclusions: OMP is a very efficient and effective teaching tool that has been used in clinical departments for teaching students for quite some time, but its use in paraclinical departments is limited. Some paraclinical departments are still not familiar with this method and need an introduction to this student-friendly and learner-centered teaching method. It can be used for Pathology slide teaching even during busy reporting days.

## Introduction

One-minute preceptor (OMP) is one of the effective teaching tools that comprises five micro-skills. This model was introduced by Neher et al. in 1992 [1]. It is commonly used in clinical Outpatient department (OPD) settings as a teaching tool and is a preferred method among students [2-3]. It is a concise method of teaching which is time-efficient and learner-centered, with feedback being one of the important components [4].

When it was first introduced, it comprised five micro-skills. The cognitive domain is addressed by the first two micro-skills, while the feedback component is covered by the next three micro-skills. The sixth skill, which promotes self-directed learning among students, was added later on [5]. The six micro-skills included in OMP are as follows: get a commitment, probe for supporting evidence, teach general rules, reinforce what was done well, correct errors, and identify the next learning step [5-6].

The traditional way of teaching slides to pathology postgraduate (PG) students includes slide teaching during daily routine reporting and slide seminars in which a set of slides is provided to the PG students, followed by observation of each slide under a microscope, reaching the differential diagnosis/diagnosis, which is followed by slide discussion with faculty. During busy days, when lots of case slides are to be reported, the routine teaching by faculty usually includes showing only the positive findings in the slides to the PG residents. This compromises the aspect of logical thinking and reasoning in students, and the lack of feedback compromises academic improvement. 

The problem here is the time constraints imposed by the faculty during busy reporting days, as discussing each slide in detail during reporting is time-consuming. This can be resolved by introducing OMP as a teaching method in the routine slide teaching. Being a concise method, it can be easily incorporated into routine slide teaching [5,7]. The feedback component and Self-directed learning further enhance learning by the students as they get immediate feedback on a particular topic and are motivated to read more about it. Its use in Clinical settings has become quite common. It has been used for teaching students in various clinical and even paraclinical subjects like Pediatrics, Obstetrics, and Gynecology, Surgery, Pulmonary Medicine, Orthopedics, ENT, and Anatomy [3,5,8-12]. It is even being used for teaching nursing students [13].

Recent studies have shown that medical students prefer blended and learner-centered educational approaches rather than relying solely on conventional teaching methods. This changing educational environment highlights the need for structured interactive teaching strategies such as the OMP [14]. Although OMP has been successfully used in various clinical teaching settings, its role in PG pathology education remains less explored. Pathology slide teaching requires development of diagnostic reasoning and independent interpretation skills; however, opportunities for structured feedback and learner engagement may be limited during routine reporting. Therefore, this study was undertaken to explore the feasibility and perceptions of PG students and faculty regarding the use of OMP as a teaching-learning method (TLM) in pathology [1-2,7].

## Materials and methods

This study was conducted in the Department of Pathology, Christian Medical College and Hospital, Ludhiana.

Study design

This was an interventional educational study that was conducted over a period of three months, in which 11 pathology PG students and seven faculty members of pathology participated.

Inclusion criteria

PG students and faculty members of the Department of Pathology, Christian Medical College and Hospital, Ludhiana, who consented to participate in the study were included.

Exclusion criteria

PG students and faculty members of the Department of Pathology, Christian Medical College and Hospital, Ludhiana, who did not consent to participate in the study were excluded.

Methodology

After obtaining approval from the Institutional Research Committee (IRC Ref. CMC/3252) and Institutional Ethics Committee (IEC Ref: IECBMHR/202405-196/Apvl-AMCE/Patho), an introductory and training session on OMP was conducted for faculty and PG students of pathology. Following this, a set of seven slides was provided to the PG students seven days before the OMP sessions. One OMP per student was conducted by seven faculty members. In this way, each faculty member conducted one OMP session with each of the 11 PG students, and each PG student had seven OMP sessions with the faculty. Each OMP session lasted approximately 7-8 minutes. The discussion slides included seven peripheral blood smears with a diagnosis of Chronic Myeloid Leukemia, Chronic Lymphocytic Leukemia, Hairy Cell Leukemia, Acute Myeloid Leukemia, Acute Lymphoblastic Leukemia, Megaloblastic Anemia, and Iron Deficiency Anemia. Traditional slide teaching routinely practiced in the department served as the internal comparator for participant perceptions. Although no separate randomized control group was established, participants’ perceptions were based on direct comparison with conventional teaching.

After obtaining consent, a feedback questionnaire, validated by medical education experts for content relevance and clarity, was administered to PG students and faculty using a five-point Likert scale on Google Form. In addition, semi-structured in-depth interviews were also taken for them. The data collected were recorded in Microsoft Excel sheets and transferred to SPSS software for statistical analyses of quantitative data. The Satisfaction Index (SI) was calculated for the perceptions of PG students and faculty using the formula described by Kaur et al. [15]

\[ SI(\%) = \frac{(n_1 \times 1) + (n_2 \times 2) + (n_3 \times 3) + (n_4 \times 4) + (n_5 \times 5)}{N \times 5} \times 100 \] where $n_1$-$n_5$ represent the frequencies of responses for each Likert scale category and *N* represents the total number of respondents.

Reliability testing for questionnaires was done using Cronbach’s alpha. Semi-structured in-depth interviews were conducted for three PG students and three faculty members after completion of the OMP sessions to explore their perceptions and experiences regarding OMP in pathology teaching. Participants were selected purposively to obtain views from different years of residency and faculty positions. Each interview lasted approximately 15-20 minutes and was conducted in a quiet departmental setting after obtaining informed consent. All interviews were audio-recorded. Participants were encouraged to openly share their experiences and perceptions regarding the OMP method.

After completion of data collection, all audio recordings were transcribed verbatim into English and checked for accuracy and completeness. The transcripts were then imported into NVivo software (version 15; Lumivero, Denver, CO) for coding and thematic analysis. Repeated reading of transcripts was performed to improve familiarity with the data and ensure accurate interpretation of participant responses. Data analysis was carried out using a thematic approach. Initial open coding was performed to identify recurring ideas, perceptions, and experiences from the transcripts. Similar codes were grouped into code families and later organized into subthemes and broader themes based on common meanings and patterns. Coding and interpretation were performed simultaneously to preserve participant perspectives and contextual meaning throughout the analysis process.

## Results

This study included 11 postgraduate (PG) students and seven faculty members from the Department of Pathology. Among the PG students, four (36.4%) were first-year, three (27.3%) were second-year, and four (36.4%) were third-year students. Of the 11 PG students, 10 (90.9%) were female and one (9.1%) was male. Among the faculty members, six (85.7%) were female and one (14.3%) was male. The faculty comprised two (28.6%) professors, three (42.9%) associate professors, and two (28.6%) assistant professors.

The questionnaire was pilot-tested among a small group of PG students to assess clarity, ease of response, and face validity. Minor revisions were made based on participant feedback. Reliability analysis demonstrated excellent internal consistency for both the faculty and PG student questionnaires (Cronbach’s alpha = 0.91). Item analysis showed satisfactory performance across domains, although one student questionnaire item (“OMP is less threatening than traditional teaching”) exhibited comparatively weaker alignment. The questionnaire items were developed based on the established principles of the OMP model. Overall, the questionnaire was found to be a reliable instrument for assessing the perceptions of OMP among PG students and faculty.

The perceptions and satisfaction indices of PG students and faculty members are shown in Tables 1-2, respectively.

**Table 1:** Perceptions of faculty regarding slide teaching using the One-Minute Preceptor (OMP) in pathology. Responses were recorded on a five-point Likert scale (1 = Strongly Disagree, 2 = Disagree, 3 = Neither Agree nor Disagree, 4 = Agree, 5 = Strongly Agree). Satisfaction Index (SI%) was calculated based on the overall response distribution.

Sr. no.	Statement	Strongly disagree (1), *n* (%)	Disagree (2), *n* (%)	Neither agree nor disagree (3), *n* (%)	Agree (4), *n* (%)	Strongly agree (5), *n* (%)	Satisfaction Index (SI%)
1	The introductory session on OMP helped me to understand OMP as a teaching method.	0	0	0	2 (28.6)	5 (71.4)	94.3
2	I enjoyed the slide teaching using OMP.	0	0	0	2 (28.6)	5 (71.4)	94.3
3	OMP improves the interaction between students and teachers.	0	0	0	0	7 (100)	100
4	I am satisfied with the use of OMP as a teaching method.	0	0	1 (14.3)	2 (28.6)	4 (57.1)	88.5
5	OMP helps to increase the reasoning and analytical skills of students.	0	0	0	2 (28.6)	5 (71.4)	94.2
6	OMP helps to inculcate the habit of reaching a provisional diagnosis in students.	0	0	0	3 (42.9)	4 (57.1)	91.3
7	I got an opportunity to give feedback during OMP.	0	0	0	2 (28.6)	5 (71.4)	94.2
8	OMP motivates students toward self-directed learning.	0	0	1 (14.3)	2 (28.6)	4 (57.1)	88.5
9	I am motivated to use OMP as a teaching method for postgraduate pathology residents.	0	0	0	2 (28.6)	5 (71.4)	94.3
10	OMP should be included in pathology postgraduate teaching as a regular teaching method.	0	0	1 (14.3)	1 (14.3)	5 (71.4)	91.4

**Table 2:** Perceptions of postgraduate students regarding slide teaching using the one-minute preceptor (OMP) in pathology. Responses were recorded on a five-point Likert scale (1 = Strongly Disagree, 2 = Disagree, 3 = Neither Agree nor Disagree, 4 = Agree, 5 = Strongly Agree). Satisfaction Index (SI%) was calculated based on the overall response distribution.

Sr. no.	Statement	Strongly disagree (1), *n* (%)	Disagree (2), *n* (%)	Neither agree nor disagree (3), *n* (%)	Agree (4), *n* (%)	Strongly agree (5), *n* (%)	Satisfaction Index (SI%)
1	An introductory session on OMP helped me to understand OMP as a teaching method.	0	0	0	5 (45.5)	6 (54.5)	90.9
2	I enjoyed the slide teaching using OMP.	0	0	0	3 (27.3)	8 (72.7)	94.5
3	I am satisfied with the use of OMP in Pathology postgraduate teaching.	0	0	0	5 (45.5)	6 (54.5)	90.9
4	OMP helped to build my confidence.	0	0	0	5 (45.5)	6 (54.5)	90.9
5	OMP improved my presentation skills.	0	0	0	4 (36.4)	7 (63.6)	92.7
6	OMP helped to increase my reasoning and analytical skills in slide diagnosis.	0	0	0	5 (45.5)	6 (54.5)	90.9
7	OMP helped me to inculcate the habit of reaching a provisional diagnosis.	0	0	0	4 (36.4)	7 (63.6)	92.7
8	Feedback during OMP helped me to improve my diagnostic skills.	0	0	0	4 (36.4)	7 (63.6)	92.7
9	OMP motivated me toward self-directed learning.	0	0	0	5 (45.5)	6 (54.5)	90.9
10	OMP is less threatening than the traditional method of teaching.	0	0	0	6 (54.5)	5 (45.5)	89.1
11	OMP should be included in pathology postgraduate teaching as a regular teaching method.	0	0	1 (9.1)	5 (45.5)	5 (45.5)	87.2

Semi-structured, in-depth interviews

After the conduct of the OMP sessions, PG students and faculty members were interviewed regarding their perceptions of OMP in pathology PG teaching. Tables 3-4 show the results of the semi-structured, in-depth interviews.

**Table 3:** Thematic analysis of in-depth interviews of postgraduate students. Thematic analysis was performed using responses obtained from in-depth interviews with postgraduate students. Representative quotes are presented verbatim to support the identified themes. OMP, one-minute preceptor

Theme	Description/Findings	Representative quote
New experience	Residents came to know about OMP during the introductory session and were informed regarding its goals and objectives.	“We had a presentation in the department in which I was able to learn about OMP and I was completely informed about the goals and objectives of OMP in that presentation.”
Comparison with traditional method	Residents perceived OMP to be better than traditional slide teaching because of one-to-one interaction, improved learning, and diagnosis-based slide interpretation.	“This method is better than traditional teaching as it promotes a diagnosis-based approach to slide interpretation through individual discussion with the consultant, from patient history to final diagnosis, making learning more effective.”
Peer acceptance	Residents felt that OMP was well accepted among peers and recommended its adoption in other courses.	“Yes, I have discussed it among my peers. Gradually everyone will accept it… I will definitely recommend it to be adopted in other similar courses.”
Teaching-learning support for OMP	Residents reported receiving adequate support and constructive guidance from faculty during OMP sessions.	“The faculty were supportive and provided constructive feedback on both positive and negative aspects of the cases, making the discussions helpful and guidance - oriented.”
Scheduling for OMP	Residents suggested preparing regular schedules/rosters for sustaining OMP sessions in the department.	“Receiving slides regularly and discussing them through the OMP approach will help sustain this practice in the department.”

**Table 4:** Thematic analysis of in-depth interviews of faculty. Thematic analysis was performed using responses obtained from in-depth interviews with faculty members. Representative quotes are presented verbatim to support the identified themes. OMP, one-minute preceptor

Theme	Description/Findings	Representative quote
A new teaching-learning method	Faculty members stated that they became aware of OMP only during the introductory session of the study.	“I came to know about this OMP teaching method from the introductory lecture which was conducted in the department of Pathology.”
Student-teacher interaction and feedback	Faculty perceived OMP as an effective method because of one-to-one interaction, immediate feedback, and promotion of self-directed learning.	“It is an effective method as it allows one-to-one interaction, immediate feedback, and guidance for self-directed learning, helping students improve academically and develop better learning skills.”
Motivation	Faculty expected OMP to motivate students toward self-directed learning, improve diagnostic approach, and build confidence.	“By this method, students get motivated to study on their own, clarify their doubts, and learn the correct approach to interpreting a slide, which is more important.”
Duration of teaching-learning encounter	Faculty felt that OMP requires extra time for conduction because of individualized discussions.	“The main problem with this method is that it is time-consuming, as extra time has to be taken out for each resident.”
Temporal placement	Faculty suggested that OMP should be implemented throughout all three years of pathology residency.	Faculty stated that OMP should be implemented in all three years of pathology residency.
Students response	Faculty perceived that students were receptive to OMP and came prepared for discussions.	“The students were receptive, came prepared after reading the slides, and the discussions helped identify and correct areas where they were lacking.”
Scheduling of OMP	Faculty recommended preparing rosters/schedules for regular implementation of OMP sessions.	“OMP can be promoted in the department by preparing a roster where faculty members are assigned specific topics on a weekly or biweekly basis.”

## Discussion

During busy reporting days, the regular slide discussion between the faculty and the pathology PG students gets compromised because of time constraints. As teachers are under pressure to complete the reporting workload, they frequently provide only the diagnosis without detailed discussion. This limits students’ analytical thinking, reduces active participation in slide interpretation, and minimizes opportunities for feedback. OMP addresses this gap by encouraging learners to independently interpret slides, justify their diagnosis, and receive immediate feedback. Similar observations were reported by Dandekar et al., who emphasized that OMP promotes active learning and clinical reasoning even in time-constrained settings. Still, many medical teachers and students are unaware of this TLM [8]. This was also observed in the present study, where all PG students and faculty stated that they learned about OMP only in the introductory session and were previously unaware of it. In our study, all participating faculty members perceived OMP as a useful and satisfactory TLM.

PG students perceived that OMP helped to build their confidence and improved their reasoning, analytical, diagnostic, and presentation skills while also motivating self-directed learning. Similar findings were observed by Aggarwal et al. in their study on Pulmonary Medicine residents [9]. Yadav et al., in their study involving 24 orthopedic residents, also reported that residents considered OMP to be an effective teaching method that increased confidence and improved clinical skills [10].

OMP provided structured one-to-one interactions between teachers and students, allowing learners to clarify doubts, justify their diagnoses, and receive immediate feedback in a non-threatening environment. Feedback is one of the key components of OMP; positive feedback boosts learner confidence, while constructive feedback helps identify areas for improvement [8]. In the present study, all PG students agreed that feedback received during OMP improved their diagnostic skills, and both PG students and faculty identified individualized interaction as one of the most valuable aspects of the method. PG students also perceived OMP to be less threatening than traditional teaching methods. Similar findings were reported by Arya et al., who observed that constructive feedback improved resident performance, while Furney et al. and Gatewood et al. demonstrated improved learning outcomes associated with immediate feedback [3,6,13]. The interactive nature of OMP has also been appreciated by learners and teachers in other studies [11].

All the faculty were satisfied with the use of OMP and enjoyed slide teaching using this method. They reported that they became aware of OMP only during the introductory session of this study. Although OMP has been used effectively in clinical settings since 1992, many medical teachers are still unaware of this TLM [8]. This highlights the need for increased awareness and faculty training regarding OMP, particularly in paraclinical and non-clinical subjects such as pathology. Faculty members felt that OMP improves the interaction between students and teachers, which helped them identify the learning needs of individual students. Similar observations were reported by Gulati et al., where faculty feedback suggested that OMP helped identify lacunae in students’ understanding and learning [7].

In our study, all faculty members were motivated to use OMP. Although most faculty agreed that OMP should become a regular TLM in pathology, one faculty member remained neutral. This neutral response may be attributed to time constraints associated with conducting one-to-one discussions. OMP requires individualized interaction; therefore, additional time is needed to conduct sessions for multiple residents. However, faculty suggested that OMP could be effectively integrated into routine reporting sessions where fewer residents are present in each unit. Similar concerns regarding time constraints were reported by Gulati et al. in their study involving pathology residents and faculty [7]. Yadav et al. also observed that 57% of orthopedic faculty members perceived time as a limitation [10].

All faculty members (100%) agreed that OMP improves PG students’ reasoning and analytical skills and inculcates the habit of formulating differential diagnoses. Similar findings were reported by Yadav et al., where 93% of faculty agreed that OMP improved clinical skills. [10] Sharma et al. also reported that 75% of faculty believed OMP enhanced residents’ clinical reasoning skills [5]. Cheema et al. observed similar findings in interns and faculty from Obstetrics and Gynecology [12].

Faculty members also perceived that OMP helped identify gaps in students' learning and facilitated timely, individualized guidance. Similar observations were reported by Furney et al. [6].

Most faculty members (6/7; 85.7%) agreed that OMP motivated students toward self-directed learning. This may be attributed to the reflective and feedback-oriented nature of OMP, which encourages learners to explore topics independently. Gulati et al. also reported similar findings [7].

Most faculty members in the present study supported the inclusion of OMP as a regular TLM in postgraduate pathology education. Similarly, Cheema et al. reported that 83.5% of faculty recommended incorporating OMP into routine teaching practice [12].

The present study demonstrates that the OMP can be effectively adapted for postgraduate pathology teaching. It provided structured one‑to‑one discussions, immediate feedback, and opportunities for self‑directed learning even during busy reporting schedules. Both PG residents and faculty perceived OMP as a useful, satisfactory, and learner‑centered teaching method. The high satisfaction indices and strong reliability of the questionnaire further strengthen the credibility of these findings.

The positive perceptions observed in the present study also support the broader value of structured educational interventions in medical training. A recent institutional study reported that a structured communication-skills module, supported by faculty sensitization, observed assessment, and learner feedback, improved students’ confidence and perceived communication competence. Although the educational context and outcomes differed from the present pathology-based OMP intervention, this study highlights the importance of a structured, interactive, feedback-oriented teaching approach in strengthening learner engagement and competence [16].

This study was limited by its single-center design, small sample size, short duration, and absence of a control group, which restricts generalizability. In addition, the study primarily focused on perceptions and satisfaction rather than objective educational outcomes. Although this provided valuable insights into the feasibility and acceptability of OMP, future studies should evaluate its impact on diagnostic accuracy, academic performance, clinical competence, and long-term knowledge retention to establish educational effectiveness. The absence of objective pre- and post-intervention assessment, the possibility of response bias associated with self-reported questionnaires, and lack of long-term follow-up may also have influenced the findings. However, reliability testing, pilot validation, and construct validity support the robustness of the study tool.

Future studies with larger sample sizes and multicentric participation are required to further validate the effectiveness of OMP in PG pathology education. Comparative studies using objective assessment methods, such as pre- and post-intervention evaluation of diagnostic reasoning, slide interpretation skills, and academic performance, may provide better insight into its educational impact. Long-term follow-up studies may help assess knowledge retention and sustained changes in learning behavior.

## Conclusions

This study demonstrates that the OMP is a feasible, interactive, and learner-centered TLM for PG pathology education. By promoting diagnostic reasoning, immediate feedback, and self-directed learning, OMP addresses important limitations of traditional slide teaching during busy reporting schedules. Despite its established role in clinical teaching, awareness and use of OMP in pathology remain limited. The findings of this study highlight the need for greater awareness, faculty development initiatives, and incorporation of OMP into routine pathology slide teaching to promote active learning and improve PG training.
